# Metal Ion-Binding Properties of the Diphosphate Ester Analogue, Methylphosphonylphosphate, in Aqueous Solution

**DOI:** 10.1155/MBD.1999.321

**Published:** 1999

**Authors:** Bin Song, Jing Zhao, Fridrich Gregáň, Nadja Prónayová, S. Ali A. Sajadi, Helmut Sigel

**Affiliations:** 1 Institute of Inorganic Chemistry University of Basel Spitalstrasse 51 Basel CH-4056 Switzerland; 2 Faculty of Pharmacy Comenius University Kalinčiaková 8 Bratislava 83232 Slovakia; 3 Faculty of Chemistry Slovak University of Technology Radlinskeho 9 Bratislava 81237 Slovakia

## Abstract

The stability constants of the 1:1 complexes formed between methylphosphonylphosphate (MePP^3-^), CH^3^P(O)^-^_2_-O-PO3^2-^, and Mg^2+^, Ca^2+^, Sr^2+^, Ba^2+^, Mn^2+^, Co^2+^, Ni^2+^, Cu^2+^, Zn^2+^, ​ or Cd^2+^ (M^2+^) were determined by potentiometric pH titration in aqueous solution (25 °C; *l* = 0.1 M, NaNO_3_). Monoprotonated M(H;MePP) complexes play only a minor role. Based on previously established correlations for M^2+^-diphosphate monoester complex-stabilities and diphosphate monoester β-group. basicities, it is shown that the M(Mepp)^-^ complexes for Mg^2+^ and the ions of the second half of the 3d series, including Zn^2+^ and Cd^2+^, are on average by about 0.15 log unit more stable than is expected based on the basicity of the terminal phosphate group in MePP^3-^. In contrast, Ba(Mepp)^-^ and Sr(Mepp)^-^ are slightly less stable, whereas the stability for Ca(Mepp)^-^ is as expected, based on the mentioned correlation. The indicated increased stabilities are explained by an increased basicity of the phosphonyl group compared to that of a phosphoryl one. For the complexes of the alkaline earth ions, especially for Ba^2+^, it is suggested that outersphere complexation occurs to some extent. However, overall the M(Mepp)^-^ complexes behave rather as expected for a diphosphate monoester ligand.


### METAL ION-BINDING PROPERTIES OF THE DIPHOSPHATE ESTER ANALOGUE, METHYLPHOSPHONYLPHOSPHATE, IN AQUEOUS SOLUTION

Bin Song[1], Jing Zhao[1], Fridrich Gregáň[2], Nadja Prónayová[3], S. Ali A. Sajadi[1] and Helmut Sigel*[1]

[1] Institute of Inorganic Chemistry, University of Basel, Spitalstrasse 51, CH-4056 Basel, Switzerland
[2] Faculty of Pharmacy, Comenius University, Kalinčiaková 8, 83232 Bratislava, Slovakia
[3] Faculty of Chemistry, Slovak University of Technology, Radlinskeho 9, 81237 Bratislava, Slovakia

**Abstract**

The stability constants of the 1:1 complexes formed between methylphosphonylphosphate ($MePP^{3-}$), $CH_3P(O)_2^-$-O-$PO_3^{2-}$, and $Mg^{2+}$, $Ca^{2+}$, $Sr^{2+}$, $Ba^{2+}$, $Mn^{2+}$, $Co^{2+}$, $Ni^{2+}$, $Cu^{2+}$, $Zn^{2+}$, or $Cd^{2+}$ ($M^{2+}$) were determined by potentiometric pH titration in aqueous solution (25 °C; $I$ = 0.1 M, $NaNO_3$). Monoprotonated M(H;MePP) complexes play only a minor role. Based on previously established correlations for $M^{2+}$-diphosphate monoester complex-stabilities and diphosphate monoester β-group basicities, it is shown that the $M(MePP)^-$ complexes for $Mg^{2+}$ and the ions of the second half of the 3d series, including $Zn^{2+}$ and $Cd^{2+}$, are on average by about 0.15 log unit more stable than is expected based on the basicity of the terminal phosphate group in $MePP^{3-}$. In contrast, $Ba(MePP)^-$ and $Sr(MePP)^-$ are slightly less stable, whereas the stability for $Ca(MePP)^-$ is as expected, based on the mentioned correlation. The indicated increased stabilities are explained by an increased basicity of the phosphonyl group compared to that of a phosphoryl one. For the complexes of the alkaline earth ions, especially for $Ba^{2+}$, it is suggested that outersphere complexation occurs to some extent. However, overall the $M(MePP)^-$ complexes behave rather as expected for a diphosphate monoester ligand.

### 1. INTRODUCTION

Phosphonate derivatives like 9-[2-(phosphonomethoxy)ethyl]adenine (PMEA) or (*S*)-9[3-hydroxy-2-(phosphonomethoxy)propyl]adenine (HPMPA) belong to a promising class of nucleotide analogues with antiviral properties[1] and indeed, there is hope that therapeutic agents against HIV will result from this class.[2,3] $PMEA^{2-}$ and $HPMPA^{2-}$ are evidently analogues of adenosine 5'-monophosphate ($AMP^{2-}$) and 2'-deoxyadenosine 5'-monophosphate ($dAMP^{2-}$).[4,5] After twofold phosphorylation by cellular nucleotide kinases,[6] the resulting triphosphate analogues can serve as substrates for viral DNA polymerase or reverse transcriptase which subsequently terminate the growing nucleic acid chain.[2,7]

The fact that most nucleotide-relevant enzymes, like DNA and RNA polymerases, kinases and ATP synthases, are metal ion- (often $Zn^{2+}$)-dependent[8,9] and that they use nucleotides as substrates only in the form of (mostly $Mg^{2+}$) complexes[9] has led to intensive studies of the metal ion-binding properties of HPMPA[5] and especially of PMEA and its derivatives.[2,10,11] However, at this time the coordinating properties of the phosphorylated compounds are unknown. Since replacement of an O-P bond by a C-P bond makes a compound more basic, as is known for example from the properties of methyl phosphate[12] and methylphosphonate,[4] it is desirable to learn how the metal ion-binding properties change if an ester bond of a diphosphate is replaced by a C-P bond. The most simple of the latter mentioned compounds is evidently methylphosphonylphosphate, $CH_3P(O)_2^-$-O-$PO_3^{2-}$ ($MePP^{3-}$), which can be considered as a diphosphate monoester analogue.[13]

Very recently we have established the correlation between metal ion complex stability and ligand basicity for a series of structurally related diphosphate monoester ligands by constructing $\log K^{M}_{M(R\text{-}DP)}$ versus $pK^{H}_{H(R\text{-}DP)}$ plots.[14] Based on these results we are now in the position to study and to evaluate the metal ion-binding properties of methylphosphonylphosphate. We are reporting here the stability constants of the $M(MePP)^-$ complexes with $Ba^{2+}$, $Sr^{2+}$, $Ca^{2+}$, $Mg^{2+}$, $Mn^{2+}$, $Co^{2+}$, $Ni^{2+}$, $Cu^{2+}$, $Zn^{2+}$, and $Cd^{2+}$. As we shall see, in most instances a slight stability enhancement is observed which we attribute to the increased basicity of the phosphonyl group.

### 2. MATERIALS AND METHODS

### 2.1. Materials and Apparatus

Trisodium methylphosphonylphosphate (IUPAC name: phosphoric methylphosphonic monoanhydride trisodium salt) was prepared in Bratislava similarly as described recently for alkyl diphosphate esters;[15] further details are given in ref. [13].

All the other reagents were the same as used previously.[12,14] The solutions were prepared with deionized, ultrapure (MILLI-Q185 PLUS; from Millipore S. A., 67120 Molsheim, France) and $CO_2$-free water. The aqueous stock solutions of MePP were freshly prepared daily and their exact concentrations were measured each time by titrations with NaOH.

The equipment for the potentiometric pH titrations and the computers employed in the calculations were the same as recently.[12,14]

### 2.2. Determination of Acidity Constants

The determination of the acidity constants $K^H_{H_2(MePP)}$ of $H_2(MePP)^-$ was described.[13] Values for $K^H_{H(MePP)}$ of $H(MePP)^{2-}$ were determined[13] by titrating 50 mL of aqueous 0.54 mM $HNO_3$ (25 °C; $I$ = 0.1 M, $NaNO_3$) in the presence and absence of 0.3 mM MePP under $N_2$ with 1 mL of 0.03 M NaOH. The experimental data were evaluated (with a curve-fitting procedure using a Newton-Gauss nonlinear least-squares program) in the pH range 4.9 to 8.0, which corresponds to about 2% and 96% neutralization, respectively, for the equilibrium $H(MePP)^{2-}/MePP^{3-}$. The final result is the average of 20 independent pairs of titrations.

The direct pH-meter readings were used to calculate the acidity constants; i.e., these constants are so-called practical, mixed or Brønsted constants.[16] Their negative logarithms given for aqueous solutions at $I$ = 0.1 M ($NaNO_3$) and 25 °C may be converted into the corresponding concentration constants by subtracting 0.02 from the listed p$K_a$ values;[16] this conversion term contains both the junction potential of the glass electrode and the hydrogen ion activity.[16,17] No conversion is necessary for the stability constants of the metal ion complexes.

### 2.3. Determination of Stability Constants

The conditions for the estimation and determination of the stability constants $K^M_{M(H;MePP)}$ and $K^M_{M(MePP)}$ of the M(H;MePP) and $M(MePP)^-$ complexes, respectively, (where $M^{2+}$ = $Mg^{2+}$, $Ca^{2+}$, $Sr^{2+}$, $Ba^{2+}$, $Mn^{2+}$, $Co^{2+}$, $Ni^{2+}$, $Cu^{2+}$, $Zn^{2+}$, or $Cd^{2+}$), were the same as given above for the acidity constant $K^H_{H(MePP)}$, i.e., 50 mL of aqueous 0.54 mM $HNO_3$ and $NaNO_3$ were titrated in the presence and absence of 0.3 mM MePP under $N_2$ with 1 mL of 0.03 M NaOH, but $NaNO_3$ was partially replaced by $M(NO_3)_2$ (25 °C; $I$ = 0.1M). The $M^{2+}$/MePP ratios used in the experiments were 1:1 and 1:2 for all systems. In the case of $Ca^{2+}$, $Sr^{2+}$, and $Ba^{2+}$, because of the low stability of the complexes, also the ratios 4:1, 10:1 and 15:1 were employed. The results were independent of the excess of $M^{2+}$ used.

Under the above given conditions, for the stability constants of the protonated M(H;MePP) complexes (the formation degree of which is small) only estimates could be obtained, which are in part also based on our previous experience with related ligands (for details see ref. [14] and also footnote [22] regarding $Ba(MePP)^-$ given in Section 3.3). These estimates were then kept fixed and the stability constant $K^M_{M(MePP)}$ was calculated for each pair of titrations with a curve-fitting procedure by taking into account the species $H^+$, $H_2(MePP)^-$, $H(MePP)^{2-}$, $MePP^{3-}$, $M^{2+}$, M(H;MePP), and $M(MePP)^-$.[13,14] The experimental data were collected every 0.1 pH unit from the lowest pH which could be reached in an experiment or from a formation degree on of about 5% for $M(MePP)^-$ to the beginning of the hydrolysis of $M(aq)^{2+}$ (e.g., with $Cu^{2+}$ or $Zn^{2+}$), which was evident from the titrations without ligand, or to a formation degree of about 85% for $M(MePP)^-$.

The final results given for the stability constants are the averages of at least three independent pairs of titrations.

### 3. RESULTS AND DISCUSSION

### 3.1. Acid-Base Properties of Twofold Protonated $MePP^{3-}$

Methylphosphonylphosphate, $CH_3P(O)_2^-$-O-$PO_3^{2-}$ can accept three protons giving $H_3(MePP)$. The first proton of this species will be released with p$K_a$ < 1.5 (cf., e.g., [12]), which means outside of the pH range of this study. For the present case the following two deprotonation equilibria have to be considered:

$$H_2(MePP)^- \rightleftharpoons H(MePP)^{2-} + H^+ \quad (1a)$$

$$K^H_{H_2(MePP)} = [H(MePP)^{2-}][H^+]/[H_2(MePP)^-] \quad (1b)$$

$$H(MePP)^{2-} \rightleftharpoons MePP^{3-} + H^+ \quad (2a)$$

$$K^H_{H(MePP)} = [MePP^{3-}][H^+]/[H(MePP)^{2-}] \quad (2b)$$

The corresponding acidity constants were determined by potentiometric pH titrations; the results are summarized in Table 1 together with some related data.

**Table 1.** Negative Logarithms of the Acidity Constants of $H_2(MePP)^-$ (eqs (1) and (2)) and of Some Related Protonated Phosphonate and Phosphate Ligands (L) as Determined by Potentiometric pH Titrations in Aqueous Solutions at 25 °C and $I$ = 0.1 M ($NaNO_3$)[a]

| Acid: $H_2L$ or $H_3L$ | $pK^H_{H_3L}$ | $pK^H_{H_2L}$ | $pK^H_{HL}$ |
|---|---|---|---|
| $CH_3P(O)(OH)_2$[4] | | 2.10±0.03[b] | 7.53±0.01 |
| $CH_3OP(O)(OH)_2$[12] | | 1.1 ±0.2 | 6.36±0.01 |
| $CH_3P(O)(OH)$-O-$P(O)(OH)_2$ (= $H_3(MePP)$) | <1.5 | 1.85±0.03 | 6.57±0.02 |
| $CH_3OP(O)(OH)$-O-$P(O)(OH)_2$ (= $H_3(MeDP)$)[14] | <1.5 | 1.62±0.09 | 6.37±0.02 |

[a] So-called practical (or mixed) constants[16] are listed; see also Section 2.2. The error limits given are *three times* the standard error of the mean value or the sum of the probable systematic errors, whichever is larger. The values in the third row are identical with those published previously.[13]
[b] See ref. [18].

As one might expect, replacement of an O-P bond by a C-P bond is most pronounced if it concerns the P atom in the vicinity of which also the acid-base reaction takes place. Indeed, from the first two entries in Table 1, which refer to the protonated forms of methylphosphonate and methyl phosphate, it is evident that both acidity constants are strongly affected: Replacement of the electron-withdrawing $CH_3O$ group by a $CH_3$ group leads to an increase of the $pK_a$ values by about 1 to 1.2 $pK$ units. The same replacement in methyl diphosphate leads to a similar but much smaller effect; i.e., $\Delta pK_a \approx 0.2$, for the difference between the $pK_a$ values of $H_2(MePP)^-$ and $H_2(MeDP)^-$ or $H(MePP)^{2-}$ and $H(MeDP)^{2-}$.

In $H_2(MePP)^-$ one proton is certainly bound at the terminal β-phosphate group, whereas the other proton could in principle either be located at the phosphonyl or also at the terminal phosphate group. Since, as we have seen above, replacement of an O-P bond by a C-P bond increases mainly the basicity of the corresponding group, the dominating tautomer of $H_2(MePP)^-$ is expected to be $CH_3P(O)(OH)$-O-$P(O)_2(OH)^-$.

### 3.2. Stability Constants of M(H;MePP) and M(MePP)$^-$ Complexes

The experimental data of the potentiometric pH titrations may be completely described by considering equilibria (1) and (2) as well as (3) and (4), provided the evaluation is not carried into the pH range where hydroxo complexes form:

$$M^{2+} + H(MePP)^{2-} \rightleftharpoons M(H;MePP) \quad (3a)$$

$$K^M_{M(H,MePP)} = [M(H;MePP)]/([M^{2+}][H(MePP)^{2-}]) \quad (3b)$$

$$M^{2+} + MePP^{3-} \rightleftharpoons M(MePP)^- \quad (4a)$$

$$K^M_{M(MePP)} = [M(MePP)^-]/([M^{2+}][MePP^{3-}]) \quad (4b)$$

As the acidity of $H_2(MePP)^-$ is rather pronounced and (therefore) the formation degree of M(H;MePP) is rather low (Section 2.3), equilibria (1) and (3) only play a minor role in the evaluation. Of course, the acidity constant of the related equilibrium (5a) may be calculated with equation (6):

$$M(H;MePP) \rightleftharpoons M(MePP)^- + H^+ \quad (5a)$$

$$K^H_{M(H,MePP)} = [M(MePP)^-][H^+]/[M(H;MePP)] \quad (5b)$$

$$pK^H_{M(H,MePP)} = pK^H_{H(MePP)} + \log K^M_{M(H,MePP)} - \log K^M_{M(MePP)} \quad (6)$$

The constants for eqs (3b), (4b), and (5b) are listed in columns 2, 3, and 4 of Table 2, respectively. To the best of our knowledge none of these constants has been determined before.[19]

Since all the acidity constants, $pK^H_{M(H,MePP)}$ = 3.3 to 5.5 (Table 2, column 4), of the M(H;MePP) complexes are lower than those of the $H(MePP)^{2-}$ species, $pK^H_{H(MePP)}$ = 6.57 (Table 1), but also significantly larger than those of $H_2(MePP)^-$, $pK^H_{H2(MePP)}$ = 1.85, it is clear that the metal ions must reside at the phosphonyl phosphate residue and the proton at the terminal phosphate group. As far as the structure of the $M(MePP)^-$ complexes is concerned, there can be no doubt that they exist as chelates, a formal structure of which is shown at the left (see also the comment regarding $Ba(MePP)^-$ and outersphere complexation in Section 3.3). It is interesting to note that the order of the stabilities of the $M(MePP)^-$ complexes does not strictly follow the common Irving-Williams sequence, but instead they follow the order now repeatedly observed[12,20] for the stabilities of phosphate metal ion complexes, i.e., $Ba^{2+} < Sr^{2+} < Ca^{2+} < Mg^{2+} < Ni^{2+} < Co^{2+} < Mn^{2+} < Cu^{2+} > Zn^{2+} < Cd^{2+}$.

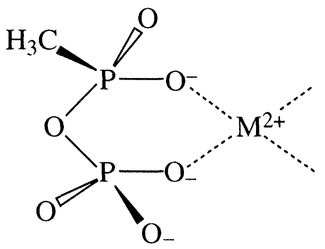

**Table 2.** Logarithms of the Stability Constants of M(H;MePP) (eq.(3)) and $M(MePP)^-$ Complexes (eq.(4)) as Estimated[a] and Determined,[b] Respectively, by Potentiometric pH Titrations in Aqueous Solutions, Together with the Negative Logarithms of the Acidity Constants (eqs (5) and (6)) of the Corresponding M(H;MePP) Complexes at 25 °C and $I$ = 0.1 M ($NaNO_3$)[c]

| $M^{2+}$ | $\log K^M_{M(H,MePP)}$ [a] | $\log K^M_{M(MePP)}$ [b] | $pK^H_{M(H,MePP)}$ [b] |
|---|---|---|---|
| $Ba^{2+}$ | 1.1 | 2.21±0.05 | 5.45±0.3 |
| $Sr^{2+}$ | 1.2 | 2.32±0.03 | 5.45±0.3 |
| $Ca^{2+}$ | 1.5 | 2.97±0.02 | 5.1 ±0.3 |
| $Mg^{2+}$ | 1.6 | 3.46±0.03[d] | 4.7 ±0.3 |
| $Mn^{2+}$ | 2.3 | 4.42±0.02[d] | 4.45±0.3 |
| $Co^{2+}$ | 2.0 | 3.98±0.04 | 4.55±0.3 |
| $Ni^{2+}$ | 2.2 | 3.78±0.02 | 5.0 ±0.3 |
| $Cu^{2+}$ | 2.4 | 5.66±0.04 | 3.3 ±0.3 |
| $Zn^{2+}$ | 2.3 | 4.46±0.03[d] | 4.4 ±0.3 |
| $Cd^{2+}$ | 2.5 | 4.60±0.02 | 4.45±0.3 |

[a] The error limits of these estimates are estimated as ±0.3 log units (see also table 2 in [14], Section 2.3, and footnote [22] regarding $Ba(MePP)^-$ in Section 3.3).
[b] The errors given are three times the standard errors of the mean value or the sum of the probable systematic errors whichever is larger. The error limits of the derived data, in the present case for column 4, were calculated according to the error propagation after Gauss.
[c] The values listed for the $Cu^{2+}$/MePP system appear also in table 1 of [13].
[d] These three values are also given in footnote 13(a) of [21].

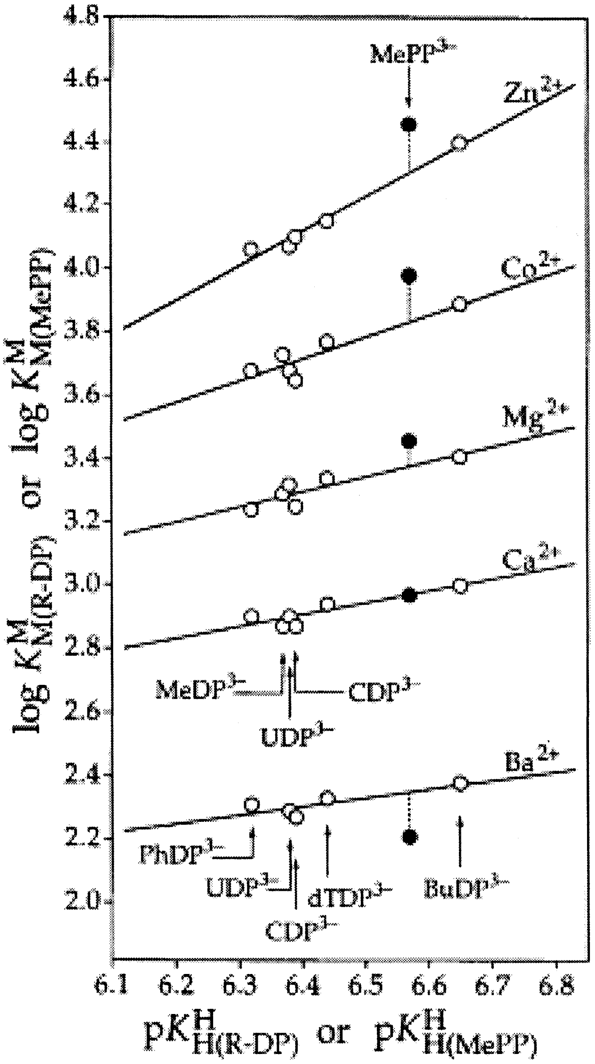

**Figure.** Comparison of the stabilities of $M(MePP)^-$ complexes (●) with those of $M^{2+}$ complexes formed with diphosphate monoesters ($R\text{-}DP^{3-}$) (○) based on the relationship between log $K^M_{M(R\text{-}DP)}$ and $pK^H_{H(R\text{-}DP)}$ for the $Ba^{2+}$, $Ca^{2+}$, $Mg^{2+}$, $Co^{2+}$, and $Zn^{2+}$ 1:1 complexes of phenyl diphosphate ($PhDP^{3-}$), methyl diphosphate ($MeDP^{3-}$), uridine 5'-diphosphate ($UDP^{3-}$), cytidine 5'-diphosphate ($CDP^{3-}$), thymidine (= 1-(2'-deoxy-β-*D*-ribofuranosyl)thymine) 5'-diphosphate ($dTDP^{3-}$), and *n*-butyl diphosphate ($BuDP^{3-}$) (from left to right). The least-squares lines are drawn through the indicated six (in the case of $Ba^{2+}$ and $Zn^{2+}$ five) data sets; the corresponding straight-line equations are given in table 4 of ref. [14]. The equilibrium constants for the $M^{2+}$/MePP systems are taken from Tables 1 and 2. All the plotted equilibrium constant values refer to aqueous solutions at 25 °C and $I$ = 0.1 M ($NaNO_3$).

### *3.3. Evaluation of the Stability of the $M(MePP)^-$ Complexes*

Very recently we have established correlations for $M^{2+}$-diphosphate monoester complex stabilities and diphosphate monoester β-group basicities.[14] A few examples of the corresponding straight-line plots are shown in the Figure into which also the corresponding data points for the $Ba^{2+}$, $Ca^{2+}$, $Mg^{2+}$, $Co^{2+}$, and $Zn^{2+}$ complexes of $MePP^{3-}$ are inserted (solid points). It is evident that in three out of the five examples the $M(MePP)^-$ complexes are somewhat more stable than is expected on the basis of the basicity of $MePP^{3-}$, i.e., on $pK^{H}_{H(MePP)}$ = 6.57, whereas the data point for the $Ca^{2+}$ system fits on the reference line and the one for the $Ba^{2+}$ system is below.

A more rigorous evaluation of this observation is possible because the straight-line equations for the log $K^{M}_{M(R-DP)}$ versus $pK^{H}_{H(R-DP)}$ plots for all ten metal ions considered in this study have been defined (see table 4 in [14]). Consequently, with a known $pK_a$ value of a monoprotonated diphosphate monoester one can calculate the stability of its corresponding $M(R-DP)^{-}$ complex. For the present case this means that we are in the position to compare the measured (exptl) stability constants with those calculated (calcd) according to the indicated procedure.

This comparison is done best by defining the stability difference expressed in equation (7):

$$\log \Delta = \log K^{M}_{M(MePP)/exptl} - \log K^{M}_{M(MePP)/calcd} \quad (7)$$

The corresponding results are summarized in Table 3.

**Table 3.** Comparison of the Stability Constants of $M(MePP)^{-}$ Complexes (eq. (4)) as Determined by Potentiometric pH Titrations (exptl)[a] in Aqueous Solution with the Corresponding Calculated Stability Constants (calcd)[b] Based on the Basicity of the Terminal Phosphate Group of $MePP^{3-}$ (25 °C; $I$ = 0.1 M, $NaNO_3$), Together with the Resulting Stability Difference log $\Delta$ (eq.(7))

| $M^{2+}$ | log $K^{M}_{M(MePP)}$ | | log $\Delta$ [c] |
|---|---|---|---|
| | exptl[a] | calcd[b] | |
| $Ba^{2+}$ | 2.21±0.05 | 2.35±0.03 | −0.14±0.06 |
| $Sr^{2+}$ | 2.32±0.03 | 2.40±0.04 | −0.08±0.05 |
| $Ca^{2+}$ | 2.97±0.02 | 2.97±0.03 | 0.00±0.04 |
| $Mg^{2+}$ | 3.46±0.03 | 3.38±0.03 | 0.08±0.04 |
| $Mn^{2+}$ | 4.42±0.02 | 4.26±0.03 | 0.16±0.04 |
| $Co^{2+}$ | 3.98±0.04 | 3.83±0.05 | 0.15±0.06 |
| $Ni^{2+}$ | 3.78±0.02 | 3.66±0.06 | 0.12±0.06 |
| $Cu^{2+}$ | 5.66±0.04 | 5.49±0.04 | 0.17±0.06 |
| $Zn^{2+}$ | 4.46±0.03 | 4.30±0.03 | 0.16±0.04 |
| $Cd^{2+}$ | 4.60±0.02 | 4.43±0.03 | 0.17±0.04 |

[a] These values are from column 3 in Table 2.
[b] Calculated with $pK^{H}_{H(MePP)}$= 6.57 (Table 1) and the straight-line equations given in table 4 of ref. [14].
[c] Regarding the error limits see footnote *b* of Table 2.

It is evident that the $M(MePP)^{-}$ complexes for $Mg^{2+}$ and especially for the metal ions of the second half of the 3d series, including $Zn^{2+}$ and $Cd^{2+}$, are on average by about 0.15 log unit more stable than is expected on the basis of the basicity of the terminal phosphate group in $MePP^{3-}$. It appears to be logic to attribute this increased stability to the higher basicity of the phosphonyl group, compared to that of a phosphoryl group as commonly present in a diphosphate monoester ligand.

Based on this explanation one wonders why the stability "increase" for the alkaline earth ions is in part reversed and becomes even negative following the order $Mg^{2+}$ (log $\Delta$ = 0.08±0.04) > $Ca^{2+}$ (0.00±0.04) > $Sr^{2+}$ (−0.08±0.05) > $Ba^{2+}$ (−0.14±0.06).[22] This order is reverse to that of the ionic radii, but it parallels the hydrated radii[23] of the alkaline earth ions. Therefore, it is our belief that for $Mg^{2+}$ and the divalent ions of the 3d series, including $Zn^{2+}$ and $Cd^{2+}$, complex formation with $MePP^{3-}$ occurs overwhelmingly innersphere (cf. also [14]), whereas for $Ca^{2+}$, $Sr^{2+}$, and $Ba^{2+}$ outersphere complex formation plays an increasing role. Maybe the larger a metal ion is, the more the methyl group at the phosphorus atom affects solvation and metal ion binding.

### 4. CONCLUSIONS

The present results obtained with $MePP^{3-}$ show that replacement of the ester-phosphoryl group by a phosphonyl residue slightly affects the complex forming properties. Indeed, for the biologically most significant metal ions, i.e., $Mg^{2+}$ and $Zn^{2+}$, a small stability increase is observed. However, since this stability increase is small compared to the overall stability constants of these complexes, one may conclude that, for example, the metal ion-binding properties of monophosphorylated and diphosphorylated PMEA, i.e., the resulting chains then correspond to diphosphate and triphosphate residues, closely resemble of the parent nucleotides as far as metal ion binding at the phosphate residues in a 1:1 ratio is concerned. In accord herewith, in mixed ligand complexes $MePP^{3-}$ behaves as expected for a simple diphosphate monoester.[13,24]

However, if a 2:1 metal ion ratio is considered, the metal ion binding properties of (2'-deoxy)-adenosine 5'-triphosphate ($dATP^{4-}$/$ATP^{4-}$) are different from those of diphoshorylated PMEA, i.e. $PMEApp^{4-}$. From studies of the metal ion promoted hydrolysis of ATP it was concluded[25-27] that for a facilitated phosphoryl transfer one metal ion should be $\alpha,\beta$ coordinated and the other should be located at the terminal $\gamma$-phosphate group of the triphosphate chain. Indeed, X-ray structural studies of a kinase have confirmed this.[28] For a nucleotidyl transfer as it is catalyzed by nucleic acid polymerases the two metal ions should be in a $M(\alpha)$–$M(\beta,\gamma)$ coordination to facilitate the bond break between the $\alpha$- and $\beta$-phosphate groups.[25,26] X-ray studies confirmed also in this case that two metal ions are involved.[29,30] As far as the latter mentioned situation is concerned, $PMEApp^{4-}$ is favored over $(d)ATP^{4-}$ in accord with studies showing that $PMEApp^{4-}$ is initially a better substrate for polymerases.[31] The explanation[21] for this observation is that the participation of the ether oxygen in metal ion binding,[4,32] giving rise to 5-membered chelates, and the enhanced basicity of the phosphonate group (as shown now) favor $M(\alpha)$–$M(\beta,\gamma)$ coordination[21] in the residue,

$$R\text{-}CH_2\text{-}O\text{-}CH_2\text{-}P_\alpha(O)_2^-\text{-}O\text{-}P_\beta(O)_2^-\text{-}O\text{-}P_\gamma(O)_3^{2-},$$

of $PMEApp^{4-}$, in comparison to the situation in a common nucleoside 5'-triphosphate,

$$R\text{-}O\text{-}P_\alpha(O)_2^-\text{-}O\text{-}P_\beta(O)_2^-\text{-}O\text{-}P_\gamma(O)_3^{2-}.$$

Of course, once the PMEA moiety is incorporated in the growing nucleic acid chain, this is terminated due to the lack of a 3'-hydroxy group, thus leading to the antiviral action of PMEA.

### ACKNOWLEDGEMENTS

The competent technical assistance of Mrs. Rita Baumbusch in the preparation of this manuscript and a research grant from the Swiss National Science Foundation, as well as partial support within the COST D8 programme by the Swiss Federal Office for Education and Science are gratefully acknowledged. B. S. and J. Z. are grateful for study leaves from the Zhongshan (Sun Yatsen) University, Guangzhou, People's Republic of China.

### REFERENCES

1. Holý, A.: *Advances in Antiviral Drug Design*, Vol. 1, De Clercq, E. (ed.); JAI Press; Greenwich CT (USA); 1993, pp 179-231.
2. Balzarini, J.; Hao, Z.; Herdewijn, P.; Johns, D. G.; De Clercq, E.: *Proc. Natl. Acad. Sci. U.S.A.* (1991) **88**, 1499-1503.
3. Naesens, L.; Snoeck, R; Andrei, G.; Balzarini, J.; Neyts, J.; De Clercq, E.: *Antivir. Chem. Chemother.* (1997) **8**, 1-23.
4. Sigel, H.; Chen, D.; Corfù, N. A., Gregáň, F.; Holý, A.; Strašák, M.: *Helv. Chim. Acta* (1992) **75**, 2634-2656.
5. Song, B.; Holý, A.; Sigel, H.: *Gazz. Chim. Ital.* (1994) **124**, 387-392.
6. (a) Merta, A.; Veselý, J.; Votruba, I.; Rosenberg, I.; Holý, A.: *Neoplasma* (1990) **37**, 111-120. (b) Foster, S. A.; Černý, J.; Cheng, Y.-c.: *J. Biol. Chem.* (1991) **266**, 238-244. (c) Balzarini, J.; De Clercq, E.: *J. Biol. Chem.* (1991) **266**, 8686-8689.
7. (a) Neyts, J.; De Clercq, E.: *Biochem. Pharmacol.* (1994) **47**, 39-41. (b) Kramata, P.; Votruba, I.; Otavá, B.; Holý, A.: *Mol. Pharmacol.* (1996) **49**, 1005-1011.
8. Sigel, H.; Sigel, A.; (eds): *Interrelations among Metal Ions, Enzymes, and Gene Expression*, Vol. 25 of *Met. Ions Biol. Syst.*; M. Dekker; New York, Basel, Hong Kong; 1989, pp 1-557.
9. Mildvan, A. S.: *Magnesium* (1987) **6**, 28-33.
10. (a) Sigel, H.: *Coord. Chem. Rev.* (1995) **144**, 287-319. (b) Sigel, H.: *J. Indian Chem. Soc.* (1997) **74**, 261-271 (P. Ray Award Lecture).

11. Blindauer, C. A.; Emwas, A. H.; Holý, A.; Dvořáková, H.; Sletten, E.; Sigel, H.: *Chem. Eur. J.* (1997) **3**, 1526-1536.
12. Saha, A.; Saha, N.; Ji, L.-n.; Zhao, J.; Gregáň, F.; Sajadi, S. A. A.; Song, B.; Sigel, H.: *J. Biol. Inorg. Chem.* (1996) **1**, 231-238.
13. Song, B.; Sajadi, S. A. A.; Gregáň, F.; Prónayová, N.; Sigel, H.: *Inorg. Chim. Acta* (1998), **273**, 101-105.
14. Sajadi, S. A. A.; Song, B.; Gregáň, F.; Sigel, H.: *Inorg. Chem.* (1999) **38**, 439-448.
15. Gregáň, F.; Kettmann, V.; Novomeský, P.; Mišiková, E.: *Boll. Chim. Farmaceutico* (1996) **135**, 229-231.
16. Sigel, H.; Zuberbühler, A. D.; Yamauchi, O.: *Anal. Chim. Acta* (1991) **255**, 63-72.
17. Irving, H. M.; Miles, M. G.; Pettit, L. D.: *Anal. Chim. Acta* (1967) **38**, 475-488.
18. Sigel, H.; Da Costa, C. P.; Song, B.; Carloni, P.; Gregáň, F.: *J. Am. Chem. Soc.* (1999) **121**, 6248-6257.
19. (a) *IUPAC Stability Constants Database*, Release 3, Version 3.02; compiled by Pettit, L. D.; Powell, H. K. J.; Academic Software, Timble, Otley, W. Yorks, U. K., 1998. (b) *NIST Critically Selected Stability Constants of Metal Complexes*, Reference Database 46, Version 5.0; data collected and selected by Smith, R. M.; Martell, A. E.; U.S. Department of Commerce, National Institute of Standard and Technology, Gaithersburg (MD), U.S.A., 1998. (c) *Joint Expert Speciation System (JESS)*, Version 5.1; joint venture by Murray, K.; May, P. M.; Division of Water Technology, CSIR, Pretoria, South Africa, and School of Mathematical and Physical Sciences, Murdoch University, Murdoch, Western Australia, 1996.
20. Sigel, H.; Song, B.: *Interactions of Metal Ions with Nucleotides, Nucleic Acids, and Their Constituents*, Vol. **32** of *Met. Ions Biol. Syst.*, Sigel, A.; Sigel, H. (eds); M. Dekker; New York, Basel, Hong Kong; 1996, pp 135-205.
21. Sigel, H.; Song, B.; Blindauer, C. A.; Kapinos, L. E.; Gregáň, F.; Prónayová, N.: *Chem. Commun.* (1999) 743-744.
22. Maybe one should mention here that the lower stability of the $M(MePP)^-$ complexes of the alkaline earth ions cannot result from an error in $\log K^{M}_{M(H,MePP)}$ (see Table 2) as can easily be shown, e.g., for $Ba(MePP)^-$: Assuming an extreme error of ±0.5 log units, i.e., $\log K^{Ba}_{Ba(H,MePP)} = 1.1 \pm 0.5$, one obtains for $\log K = 1.1$, $\log \Delta = -0.14 \pm 0.06$ (as given in Table 3), for $\log K = 0.6$ (lower limit) $\log \Delta = -0.18 \pm 0.06$, and for $\log K = 1.6$ (upper limit) $\log \Delta = -0.04 \pm 0.06$. This means, even with these extreme assumptions one does not reach a positive value for $\log \Delta$ comparable to the results observed for the complexes of the 3d ions.
23. *Gmelins Handbuch der Anorganischen Chemie*, 8. völlig neu bearbeitete Auflage, Verlag Chemie GMBH Weinheim: (a) *Magnesium* (Syst.-Nr. 27), Teil A, 1952, p. 172. (b) *Calcium* (Syst.-Nr. 28), Teil A, 1957, p. 390. (c) *Strontium* (Syst.-Nr. 29), 1931, p. 40. (d) *Barium* (Syst.-Nr. 30), 1932, p. 39.
24. (a) Sajadi, S. A. A.; Song, B.; Gregáň, F.; Sigel, H.: *Bull Chem. Soc. Ethiopia* (1997) **11**, 121-130. (b) Sajadi, S. A. A.; Song, B.; Sigel, H.: *Inorg. Chim. Acta* (1998), **283**, 193-201.
25. Sigel, H.; Hofstetter, F.; Martin, R. B.; Milburn, R. M.; Scheller-Krattiger, V.; Scheller, K. H.: *J. Am. Chem. Soc.* (1984) **106**, 7935-7946.
26. Sigel, H.: *Coord. Chem. Rev.* (1990) **100**, 453-539.
27. (a) Sigel, H.: *Inorg. Chim. Acta* (1992) **198-200**, 1-11. (b) Sigel, H.: *Pure Appl. Chem.* (1998) **70**, 969-976.
28. Tari, L. W.; Matte, A.; Goldie, H.; Delbaere, L. T. J.: *Nature Struct. Biol.* (1997) **4**, 990-994.
29. (a) Pelletier, H.; Sawaya, M. R.; Kumar, A.; Wilson S. H.; Kraut, J.: *Science* (1994) **264**, 1891-1903. (b) Pelletier, H.: *Science* (1994) **266**, 2025-2026. (c) Pelletier, H.; Sawaya, M. R.; Wolfle, W.; Wilson, S. H.; Kraut, J.: *Biochemistry* (1996) **35**, 12762-12777.
30. (a) Steitz, T. A.: *Nature* (1998) **391**, 231-232. (b) Brautigam, C. A.; Steitz, T. A.: *Curr. Opinion Struct. Biol.* (1998) **8**, 54-63.
31. Holý, A.; Votruba, I.; Merta, A.; Černý, J.; Veselý, J.; Vlach, J.; Šedivá, K.; Rosenberg, I.; Otmar, M.; Hřebabecký, H.; Trávniček, M.; Vonka, V.; Snoeck, R.; De Clercq, E.: *Antiviral Res.* (1990) **13**, 295-312.
32. Blindauer, C. A.; Holý, A.; Dvořáková, H.; Sigel, H.: *J. Biol. Inorg. Chem.* (1998) **3**, 423-433.

**Received: June 17, 1999 - Accepted: July 6, 1999 -**
**Received in revised camera-ready format: August 5, 1999**

